# Capability, opportunity, and motivation: a structural equation model of hand hygiene behavior based on the COM-B framework in a Chinese hospital setting

**DOI:** 10.3389/fpubh.2026.1755052

**Published:** 2026-05-08

**Authors:** Wenjing Jiang, Xia Li, Li Cao, Yuhan Zhang, Ping Zhou, Chunli Song, Xiaoli Chen, Yiju Xie, Juan Tang

**Affiliations:** 1 Department of Nursing, Sichuan Vocational College of Health and Rehabilitation, Zigong, China; 2 Department of Hospital Infection Management, Zigong First People’s Hospital, Zigong, China; 3 Department of Critical Care Medicine, Zigong First People’s Hospital, Zigong, China; 4 Department of Infectious Diseases, Zigong First People’s Hospital, Zigong, China

**Keywords:** behavior change, COM-B model, hand hygiene, healthcare workers, mediation analysis, structural equation modeling

## Abstract

**Background:**

Healthcare-associated infections (HAIs) present a major threat to patient safety worldwide. While hand hygiene is the primary defense against HAIs, compliance among healthcare workers (HCWs) remains inadequate, highlighting a persistent “know-do” gap. The COM-B (capability, opportunity, motivation-behavior) model offers a robust theoretical framework for understanding this behavior, yet its complex mediating pathways—particularly through structural equation modeling—remain underexplored in hand hygiene research.

**Objective:**

This study aimed to assess the utility of the COM-B model in explaining hand hygiene behavior among HCWs in China, specifically quantifying the direct effects of capability, opportunity, and motivation on behavior and testing their indirect relationships through mediated pathways.

**Methods:**

A cross-sectional study was conducted in a Chinese tertiary hospital, recruiting 1,722 HCWs. Data were collected on demographic characteristics and hand hygiene behaviors using validated questionnaires based on the COM-B framework. Structural equation modeling (SEM) was employed to examine the direct and mediated effects of capability, opportunity, and motivation on hand hygiene behavior.

**Results:**

The final COM-B model demonstrated acceptable fit (CFI = 0.917, TLI = 0.903, RMSEA = 0.115). Motivation (*β* = 0.434, *p* < 0.001) and opportunity (*β* = 0.324, *p* = 0.013) had significant positive direct effects on behavior. Capability exhibited a significant negative direct effect on behavior (*β* = −0.217, *p* = 0.013), but its indirect effects via opportunity alone (*β* = 0.199, *p* = 0.017) and via the serial mediation of opportunity and motivation (*β* = 0.205, *p* = 0.001) were significant and positive. Capability primarily enhanced motivation by fostering the perception of opportunity (capability→opportunity: *β* = 0.842, *p* < 0.001; opportunity→motivation: *β* = 0.769, *p* < 0.001).

**Conclusion:**

This study validates the applicability of the COM-B model in understanding hand hygiene behavior among Chinese HCWs. Motivation is the core direct driver of behavior, while environmental opportunity acts as a critical bridge linking capability to behavior. The negative direct effect of capability suggests that isolated training interventions may have limited effectiveness without concurrent environmental support. Therefore, improving hand hygiene compliance requires multifaceted strategies that simultaneously bolster capability, optimize opportunity, and cultivate motivation.

## 1 Introduction

Healthcare-associated infections (HAIs) represent a significant global public health problem, threatening patient safety and compromising the quality of care. The World Health Organization (WHO) estimates that approximately 7% of hospitalized patients worldwide are affected by HAIs, with rates reaching 5–10% in developed countries, leading to a substantial economic burden (1). Hand hygiene is widely recognized as the most fundamental, effective, and cost-efficient measure for preventing HAIs (2, 3). Evidence demonstrates that improved hand hygiene compliance can significantly reduce HAI rates (4). Despite its established importance, hand hygiene adherence among healthcare workers (HCWs) remains suboptimal globally, with numerous studies reporting average compliance rates persisting between 40 and 60% (5, 6). This persistent “know-do” gap indicates that hand hygiene behavior is a complex phenomenon influenced by multiple factors, necessitating investigation through systematic behavioral science frameworks to identify its key determinants and inform effective intervention strategies (7).

Historically, research on hand hygiene behavior has often focused on describing compliance rates or examining the direct influence of isolated factors, such as knowledge or attitudes, with a relative lack of integrated theoretical frameworks to explain the interplay between determinants (7, 8). The COM-B (capability, opportunity, motivation-behavior) model, a comprehensive behavioral framework, posits that any behavior (B) results from the dynamic interaction of an individual’s psychological and physical capability (C), the external opportunity (O) provided by the physical and social environment, and reflective and automatic motivation (M) (9). This model has been applied to understand various health behaviors in clinical settings (10–12). However, its application to hand hygiene behavior, particularly testing its full-path model, remains relatively limited. Furthermore, existing studies in this area predominantly rely on traditional correlation or regression analyses, which are limited in their ability to simultaneously test the complex mediation pathways implied by the COM-B model and to quantitatively assess the overall goodness-of-fit between the theoretical model and empirical data (13, 14). This mismatch between the model’s complexity and methodological limitations represents a key knowledge gap.

A recent study conducted by our team in a Chinese healthcare setting provided an initial exploration of hand hygiene behavior using the COM-B model, suggesting potential mediating roles of opportunity and motivation between capability and behavior (15). This work offered preliminary insights. However, the analytical methods employed could not model the constructs as latent variables or evaluate the overall model fit.

To enable a more rigorous examination of the COM-B model’s explanatory power, this study utilized structural equation modeling (SEM). SEM allows for the simultaneous analysis of latent and observed variables within a complete theoretical model, facilitating the estimation of all hypothesized paths and providing objective indices, such as the comparative fit index (CFI) and root mean square error of approximation (RMSEA), to evaluate model fit (16, 17). The application of SEM to our data allowed for a comprehensive assessment of the proposed relationships.

Therefore, the primary aim of this study was to test the COM-B model’s applicability to hand hygiene behavior using SEM in a large sample of HCWs. The specific objectives were: (1) to evaluate the overall goodness-of-fit of the COM-B model, (2) to quantify the direct effects of capability, opportunity, and motivation on behavior, and (3) to precisely test the mediating effects of opportunity and motivation in the relationship between capability and behavior. We hypothesized that:

*H1*: The COM-B model would demonstrate an acceptable fit to the data.

*H2*: Hand hygiene opportunity and motivation would have significant positive direct effects on behavior.

*H3*: The effect of hand hygiene capability on behavior would be mediated by opportunity (Path 1) and motivation (Path 2).

*H4*: Hand hygiene capability would exert an indirect effect on behavior through a serial mediation pathway via opportunity and motivation (Path 3: capability→opportunity→motivation→behavior).

## 2 Methods

### 2.1 Study design and setting

A hospital-based cross-sectional study was conducted in May–June 2022 at a tertiary teaching hospital in Sichuan Province, China, to model the psychosocial determinants of hand hygiene behaviors among HCWs based on the COM-B framework. The study protocol was approved by the Institutional Review Board of Zigong First People’s Hospital (No: 2022031), and all procedures adhered to the principles of the Declaration of Helsinki. This study was reported following the STROBE guidelines for cross-sectional studies.

The sample size was determined *a priori* using the standard for SEM of 10–20 participants per observed variable (16). With 22 observed variables, the minimum sample size required was 220–440. Our final sample of 1,722 HCWs far exceeded this threshold, ensuring high statistical power for the analyses.

### 2.2 Participants

The HCWs were eligible for inclusion if they (1) possessed a valid medical practice qualification, (2) had a minimum of 1 year of work experience, and (3) provided informed consent to voluntarily participate in the survey. HCWs were excluded from the study if they were on leave or engaged in external training programs during the data collection period.

### 2.3 Variables and measurement instruments

#### 2.3.1 Participant characteristics

Demographic and occupational characteristics were collected via a self-administered questionnaire. The variables assessed included gender, age, education level, occupation, years of work experience, and professional title.

#### 2.3.2 The hand hygiene behavior questionnaire

The core constructs of the COM-B model (capability, opportunity, motivation) were measured using the transculturally adapted and validated Chinese version of the Hand Hygiene Behavior Questionnaire (HHBQ) developed by Tao et al. (18). The original development and validation study confirmed its robust psychometric properties, including structural validity and internal consistency. This instrument is specifically designed around the COM-B framework and comprises 17 items distributed across three corresponding subscales. All items are rated on a 5-point Likert scale ranging from 1 (strongly disagree) to 5 (strongly agree). The three subscales are detailed below:

- Hand hygiene capability was assessed using the 5-item corresponding subscale of the HHBQ (18) (see Table 1, items Cap 1–5). It measures the psychological and physical capacity to perform hand hygiene (e.g., “I know how to perform hand hygiene correctly”). In this study, the subscale showed high internal consistency (Cronbach’s *α* = 0.946).
- Hand hygiene opportunity was assessed using the 7-item corresponding subscale of the HHBQ (18) (see Table 1, items Opp 1–7). It measures the physical and social environmental factors that enable or hinder hand hygiene (e.g., “Hand hygiene facilities are conveniently located”). In this study, the subscale showed high internal consistency (Cronbach’s *α* = 0.959).
- Hand hygiene motivation was assessed using the 5-item corresponding subscale of the HHBQ (18) (see Table 1, items Mot 1–5). It measures the reflective and automatic processes that drive hand hygiene behavior (e.g., “I feel a strong sense of responsibility to perform hand hygiene”). In this study, the subscale showed high internal consistency (Cronbach’s *α* = 0.977).

**Table 1:** Measurement items for the COM-B model in hand hygiene among healthcare workers.

#	Construct and Item
Hand hygiene capability	
Cap1	I have received standardized hand hygiene training in my unit.
Cap2	I know when hand hygiene is required.
Cap3	I know how to perform hand hygiene correctly.
Cap4	I will perform hand hygiene consciously and proactively.
Cap5	I find it easy to comply with the hand hygiene requirements in my unit.
Hand hygiene opportunity	
Opp1	I have enough time to perform hand hygiene as required.
Opp2	There are sufficient hand hygiene facilities available in my unit.
Opp3	Nurses in my unit always perform hand hygiene as required.
Opp4	Physicians in my unit always perform hand hygiene as required.
Opp5	All healthcare workers who come to my unit always perform hand hygiene as required.
Opp6	The hand hygiene protocols in my unit are clear.
Opp7	There are reminders in my unit to prompt staff to perform hand hygiene.
Hand hygiene motivation	
Mot1	Staff in my unit believe that adherence to hand hygiene is important.
Mot2	The leadership in my unit emphasizes that hand hygiene compliance is important.
Mot3	I try my best to adhere to the ‘Five Moments’ for hand hygiene.
Mot4	We remind each other in my unit to perform hand hygiene.
Mot5	The infection control audits in the hospital encourage me to comply with my unit’s hand hygiene protocols.
Hand hygiene behavior	
Beh1	In the past year, I performed hand hygiene before touching a patient.
Beh2	In the past year, I performed hand hygiene before a clean/aseptic procedure.
Beh3	In the past year, I performed hand hygiene after the risk of body fluid exposure.
Beh4	In the past year, I performed hand hygiene after touching a patient.
Beh5	In the past year, I performed hand hygiene after touching patient surroundings.

#### 2.3.3 Hand hygiene behavior

Hand hygiene behavior was evaluated with a separate questionnaire based on the WHO’s “5 moments for hand hygiene” framework (6). This instrument comprises 5 items, each corresponding to one of the five critical moments (see Table 1, items Beh 1–5). Participants responded on a 5-point Likert scale regarding their frequency of compliance. The measure demonstrated good reliability in this sample, with a Cronbach’s *α* of 0.925.

### 2.4 Data collection

Data collection was conducted via an online cross-sectional survey administered on the Wenjuanxing platform (https://www.wjx.cn; also known as Questionnaire Star), a widely used online survey tool in China. Potential participants were recruited through department-based communication groups within the hospital. A survey link was disseminated through these channels, accompanied by an invitation outlining the study’s voluntary nature. Upon accessing the survey link, participants were first presented with a comprehensive information sheet detailing the study objectives, procedures, potential risks and benefits, and data confidentiality measures. Electronic informed consent was obtained from all participants before they could proceed to the questionnaire. Consent was provided by selecting an “I Agree” option after reading the information sheet. Participants were explicitly informed that they could withdraw from the study at any time without any negative consequences.

To ensure the quality and integrity of the collected data, the survey platform was configured to prevent duplicate submissions from the same IP address or device. Following the data collection phase, all responses underwent a rigorous, independent manual review by two research team members to identify and exclude invalid questionnaires. In total, 1,876 submissions were received through the survey platform. The exclusion criteria were predefined as follows: (1) exhibiting obvious logical inconsistencies (e.g., contradictory responses), (2) having over 10% of items unanswered (incomplete entries), or (3) having an implausibly short response time (less than 2 min, as determined by a pilot test). All anonymized data were stored securely on password-protected servers owned by the institution and were accessed solely by the principal investigators for research purposes, strictly adhering to the ethical principles for data confidentiality.

### 2.5 Statistical analysis

Data analysis was performed using SPSS 27.0 (IBM Corp., Armonk, NY, United States) and R software (version 4.4.3; R Foundation for Statistical Computing, Vienna, Austria). Descriptive statistics were computed using SPSS, with continuous variables presented as mean ± standard deviation and categorical variables as frequencies and percentages. Pearson correlation analysis was applied to examine bivariate relationships between normally distributed continuous variables. The internal consistency reliability of the measurement instruments was assessed using Cronbach’s alpha coefficient, with a value greater than 0.70 deemed acceptable.

Confirmatory factor analysis (CFA) and SEM were conducted using the lavaan package in R. Model fit was evaluated against the following criteria: chi-square/degrees of freedom (*χ*^2^/df) < 3.000, CFI > 0.900, Tucker–Lewis index (TLI) > 0.900, RMSEA < 0.080, and standardized root mean square residual (SRMR) < 0.080, as suggested by Hu and Bentler (17). Given the large sample size (*N* = 1722), the interpretation of RMSEA considered recommendations by Marsh et al. (19) regarding the index’s sensitivity to sample size. Modification indices (MI) were examined to identify theoretically plausible *post hoc* model adjustments, with all MI values greater than 10 documented in Supplementary Table S1.

The significance of mediation effects was tested using the percentile bootstrap method with 5,000 resamples, generating bias-corrected 95% confidence intervals (20). Mediation was considered statistically significant if the 95% confidence interval did not include zero. Model parameters were estimated using the maximum likelihood estimation method. Full information maximum likelihood (FIML) was employed to handle any missing data, as it provides unbiased parameter estimates under the assumption of data missing at random.

All statistical tests were two-sided, with the significance level set at *α* = 0.05. Path diagrams were generated using the semPlot package. Results are presented as parameter estimates, standard errors, z-values, *p*-values, and 95% confidence intervals.

## 3 Results

### 3.1 Participants’ characteristics

A total of 1,876 submissions were received. After applying the pre-specified exclusion criteria detailed in Section 2.4, 154 invalid responses were removed. Consequently, 1,722 valid questionnaires were included in the analysis, resulting in a valid response rate of 91.79% (1,722/1,876). The participants were 1722 HCWs, mainly comprising females (79.0%), individuals with bachelor’s degrees (67.5%), nursing staff (59.2%), and those with primary professional titles (50.4%) (Table 2).

**Table 2:** Participants’ characteristics.

Variable	*n*	%
Gender		
Male	356	21.0
Female	1,366	79.0
Age (years)		
18–25	373	21.7
26–30	546	31.7
31–35	402	23.3
36–40	176	10.2
≥41	225	13.1
Education level		
Technical secondary school	13	0.8
Junior college	381	22.1
Bachelor’s degree	1,163	67.5
Master’s degree or above	165	9.6
Occupation		
Physician	449	26.1
Nurse	1,020	59.2
Medical technician	253	14.7
Work experience (years)		
1 ~ 5	622	36.1
6 ~ 10	519	30.1
11 ~ 20	385	22.4
≥21	196	11.4
Professional title		
None	238	13.8
Junior	868	50.4
Intermediate	390	22.6
Senior	226	13.1

### 3.2 Descriptive statistics and correlations among the COM-B variables

Table 3 demonstrated that the mean scores for hand hygiene capability, opportunity, motivation, and behavior were 4.80 ± 0.55, 4.75 ± 0.55, 4.83 ± 0.49, and 4.88 ± 0.36, respectively, indicating consistently high levels across all COM-B constructs. Table 3 also revealed statistically significant positive correlations among these variables, with opportunity showing strong associations with both motivation (*r* = 0.844, *p* < 0.01) and capability (*r* = 0.819, *p* < 0.01). Furthermore, motivation was significantly correlated with capability (*r* = 0.770, *p* < 0.01), opportunity (*r* = 0.844, *p* < 0.01), and behavior (*r* = 0.534, *p* < 0.01).

**Table 3:** Means and standard deviation, and correlations among the COM-B variables.

Variable	1	2	3	4
1. Hand hygiene capability	1			
2. Hand hygiene opportunity	0.819**	1		
3. Hand hygiene motivation	0.770**	0.844**	1	
4. Hand hygiene behavior	0.406**	0.505**	0.534**	1
*M* ± SD	4.80 ± 0.55	4.75 ± 0.55	4.83 ± 0.49	4.88 ± 0.36

M, mean; SD, standard deviation. **p < 0.01.

### 3.3 Structural equation modeling results

#### 3.3.1 Measurement model modification and comparison

Initial CFA indicated that the theoretical measurement model required modification to achieve acceptable fit. Guided by MI, covariances between error terms of items within the same construct were added to the model, resulting in a revised model with 4 additional degrees of freedom.

The model modification led to a significant improvement in fit, as evidenced by a reduction in the chi-square statistic of Δχ^2^(4) = 1838.159 (*p* < 0.001). All fit indices showed improvement in the revised model compared to the initial model: CFI increased from 0.884 to 0.917, TLI improved from 0.868 to 0.903, and RMSEA decreased from 0.134 to 0.115. SRMR also improved from 0.050 to 0.047. Information criteria indices decreased, with the Akaike information criterion (AIC) reduced by 1830.159 and the Bayesian information criterion (BIC) reduced by 1808.222.

The fit indices for the final revised measurement model were as follows: *χ*^2^(199) = 4870.819, CFI = 0.917, TLI = 0.903, RMSEA = 0.115, SRMR = 0.047. A comprehensive comparison of fit indices between the initial and revised models is presented in Table 4.

**Table 4:** Comparison of fit indices between the initial and MI-revised measurement models.

Fit index	Initial model	MI-revised model	Change (Δ)
*χ* ^2^	6708.978	4870.819	−1838.159
df	203	199	−4
CFI	0.884	0.917	0.033
TLI	0.868	0.903	0.035
RMSEA	0.134	0.115	−0.019
SRMR	0.050	0.047	−0.003
AIC	11508.000	9677.841	−1830.159
BIC	11782.219	9973.997	−1808.222

*χ*^2^, Chi-square; df, degrees of freedom; CFI, Comparative Fit Index; TLI, Tucker–Lewis Index; RMSEA, Root Mean Square Error of Approximation; SRMR, Standardized Root Mean Square Residual; AIC, Akaike Information Criterion; BIC, Bayesian Information Criterion. The MI-Revised model was improved by incorporating theoretically plausible error covariances based on Modification Indices (MI).

The acceptable fit of the four-factor model (CFI = 0.917, TLI = 0.903) supports the discriminant validity of the four latent constructs (capability, opportunity, motivation, and behavior) as distinct but related factors within the COM-B framework, despite the high observed correlations among capability, opportunity, and motivation (see Table 3).

#### 3.3.2 Direct effects and path coefficients

Path analysis revealed the standardized direct effects among the COM-B constructs on hand hygiene behavior. Motivation had the strongest significant direct effect on behavior (*β* = 0.434, *p* < 0.001). Opportunity also showed a significant positive direct effect on behavior (*β* = 0.324, *p* = 0.013). In contrast, capability exhibited a significant negative direct effect on behavior (*β* = −0.217, *p* = 0.013).

Analysis of the interrelationships among the COM-B constructs showed that opportunity was a strong significant predictor of motivation (*β* = 0.769, *p* < 0.001). Similarly, capability had a substantial significant positive effect on opportunity (*β* = 0.842, *p* < 0.001). However, the direct path from capability to motivation was not statistically significant (*β* = 0.139, *p* = 0.088).

#### 3.3.3 Mediation effects analysis

Bootstrap analysis with 5,000 resamples was conducted to test the mediation effects. The indirect effect of capability on behavior through opportunity was significant (*β* = 0.199, *p* = 0.017). The chain mediation effect through both opportunity and motivation was also statistically significant (*β* = 0.205, *p* = 0.001). In contrast, the specific indirect effect through motivation alone was not significant (*β* = 0.044, *p* = 0.173).

**Figure 1:**
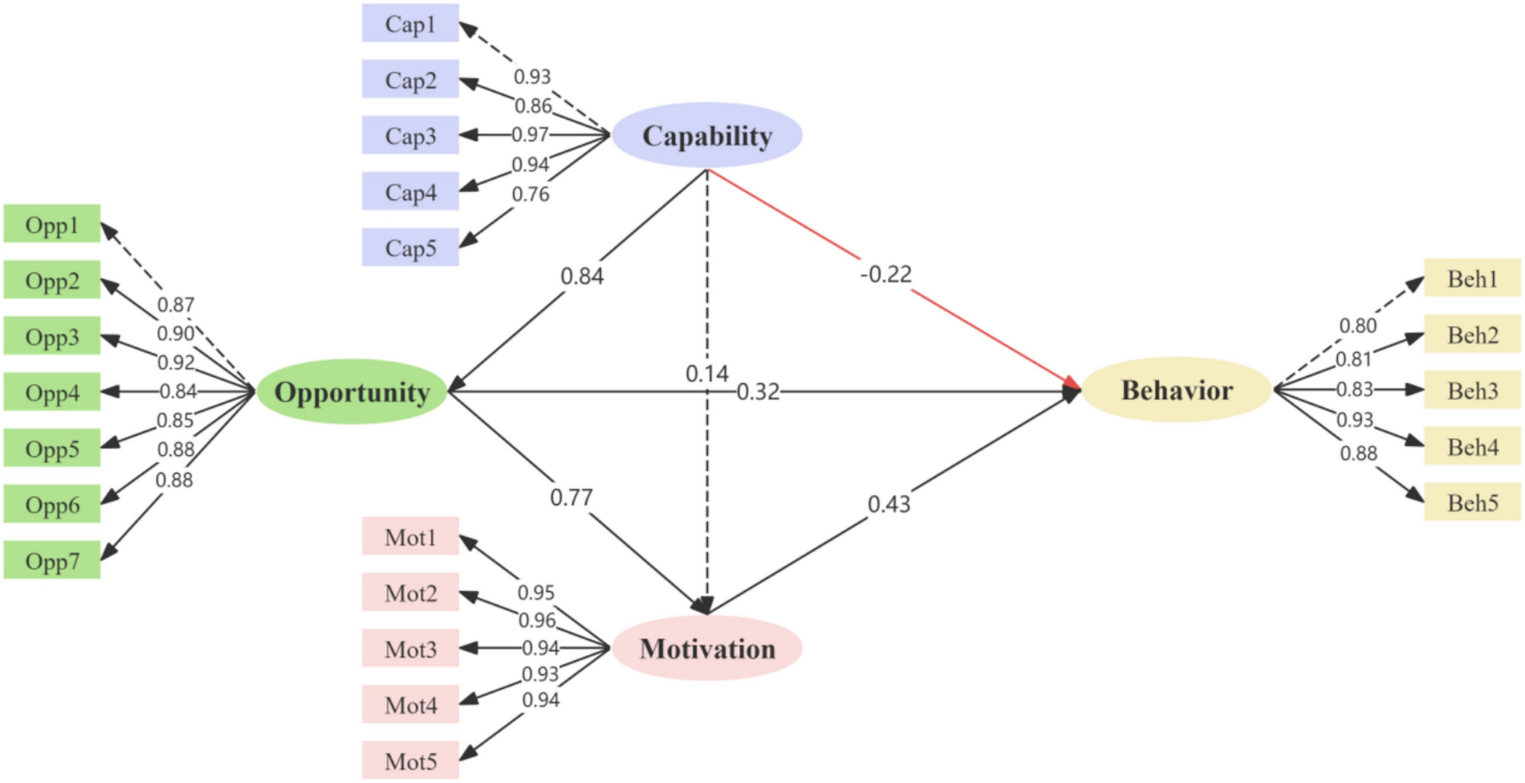
Structural equation model of the relationships between COM-B factors. Dashed lines represent paths that did not reach statistical significance at *p* < 0.05.

The total indirect effect of capability on behavior was 0.448 (*p* < 0.001), and the total effect was 0.290 (*p* < 0.001). The comprehensive results of path coefficients and mediation effects are presented in Table 5, and the complete structural model is depicted in Figure 1.

**Table 5:** Standardized path coefficients and mediation effects of the COM-B model.

Path	*β*	SE	*z*	*P*	95% CI (BCa)
Direct effects					
Behavior ← Capability (b1)	−0.217	0.064	−2.489	0.013	[−0.307, −0.058]
Behavior ← Opportunity (b2)	0.324	0.087	2.493	0.013	[0.068, 0.399]
Behavior ← Motivation (b3)	0.434	0.111	3.18	0.001	[0.190, 0.677]
Motivation ← Capability (a1)	0.139	0.073	1.707	0.088	[−0.014, 0.292]
Motivation ← Opportunity (a2)	0.769	0.071	8.873	<0.001	[0.629, 0.908]
Opportunity ← Capability (c)	0.842	0.059	15.62	<0.001	[0.776, 0.908]
Indirect Effects					
Capability → Opportunity → Behavior	0.199	0.083	2.392	0.017	[0.062, 0.373]
Capability → Motivation → Behavior	0.044	0.032	1.362	0.173	[0.003, 0.144]
Capability → Opportunity → Motivation → Behavior	0.205	0.063	3.245	0.001	[0.087, 0.333]
Total Indirect Effect	0.448	0.074	6.064	<0.001	[0.325, 0.612]
Total Effect	0.290	0.054	5.322	<0.001	[0.198, 0.415]

Model Fit Indices: *χ*^2^(199) = 4870.819, *P* < 0.001, CFI = 0.917, TLI = 0.903, RMSEA = 0.115, SRMR = 0.047. BCa, bias-corrected and accelerated bootstrap confidence interval (5,000 samples).

## 4 Discussion

This study employed SEM to rigorously test the complex mediating pathways within the COM-B framework in explaining hand hygiene behavior among HCWs in a Chinese hospital setting. Our finding that motivation and opportunity were the primary direct drivers of hand hygiene behavior is consistent with previous COM-B studies in healthcare settings (10–12, 14). This underscores that, in contexts with high baseline knowledge, activating reflective motivation and ensuring environmental support are paramount for behavioral enactment. However, the significant negative direct effect of capability contrasts with some Western studies where knowledge showed positive direct effects (7, 8), suggesting potential cultural differences in how capability translates to action. This counterintuitive finding highlights a critical theoretical nuance: when isolated from supportive conditions, higher capability alone may not lead to better performance. Our key findings support the applicability of the COM-B model and elucidate the complex interplay between its components, revealing a significant negative direct effect of capability on behavior that is counterbalanced by strong indirect effects via opportunity and motivation. The high intercorrelations observed among the three antecedent COM-B constructs (capability, opportunity, and motivation) in our study align with findings from other studies applying the COM-B model to healthcare behaviors, where these factors often show substantial covariance (21). This may reflect the model’s core premise that behavior emerges from a dynamic system of interacting components, rather than from entirely independent factors. The significant serial mediation pathway identified (capability→opportunity→motivation→behavior) provides empirical evidence for this interplay. Nevertheless, the confirmation of a better-fitting four-factor measurement model suggests that, from a measurement perspective, participants do distinguish between these concepts. These insights provide a nuanced theoretical foundation for developing targeted intervention strategies.

A pivotal and novel finding of this study is the significant negative direct effect of capability on behavior. This counterintuitive result indicates that, after accounting for the mediating roles of opportunity and motivation, a higher self-reported capability level was associated with a slight decrease in self-reported hand hygiene behavior. This pattern is consistent with a statistical suppression effect, wherein the positive total effect of capability is fully transmitted through its mediators, revealing an underlying negative direct relationship (19, 22). This phenomenon can be interpreted through the lens of cognitive dissonance or heightened sensitivity (2, 3). HCWs with advanced capability may become more acutely aware of and frustrated by environmental constraints, leading to a disconnect between knowledge and action when optimal conditions are not met (23, 24). The analysis confirms two significant mediating pathways. The first is the direct mediation through opportunity, underscoring that behavioral enactment is highly contingent on environmental support. The second, and more insightful, is the significant serial mediation path (capability→opportunity→motivation→behavior). This reveals a dynamic sequence: enhanced capability fosters the perception of a supportive environment, which in turn serves as a critical antecedent to bolstered motivation, ultimately driving behavior (25). The non-significant direct path from capability to motivation reinforces that its motivational influence is almost entirely mediated by shaping perceived opportunity. Consequently, the negative direct effect highlights a critical limitation of isolated, capability-focused training interventions, emphasizing the necessity of concurrently building a supportive environment (26, 27).

The most substantial direct driver of hand hygiene behavior was motivation. This finding strongly affirms the central proposition of the COM-B model, positioning motivation as the proximate catalyst for behavior enactment (9, 28). In a context where baseline knowledge is high, activating reflective cognitive processes—such as internalized importance and responsiveness to leadership and peer influences—becomes paramount for translating competence into action (7, 29, 30). Opportunity also exerted a significant direct positive effect, reinforcing the indispensable role of optimizing both the physical (e.g., facility availability) and social (e.g., colleague modeling) environment to reduce barriers and facilitate performance (23, 29).

The pattern of our results carries important practical implications. First, the finding that motivation is the strongest direct driver suggests that in settings like ours with high baseline knowledge, interventions should move beyond basic education to activate and sustain reflective motivation. This can be achieved through regular audit and personalized feedback, leadership endorsement, and fostering a unit culture where hand hygiene is a visible social norm. Second, the critical bridging role of opportunity underscores that improving capability alone is insufficient. Hospital administrators must concurrently ensure the easy availability of alcohol-based hand rub at the point of care, manage workloads to allow time for compliance, and streamline clinical workflows to reduce environmental barriers. Finally, the negative direct effect of capability serves as a crucial warning: investing in training without providing the necessary environmental and motivational support may yield limited or even counterproductive results. Therefore, sustainable improvement requires integrated, system-wide strategies that simultaneously address all three COM-B components.

When contextualized within existing literature, our findings offer both confirmation and extension. The independent importance of opportunity and motivation is consistent with prior studies (10–12, 14). However, by employing SEM, we move beyond correlation and regression to simultaneously test the full nomological network of the COM-B model. Our results corroborate and quantitatively test the proposition by Keyworth et al. (21) that COM-B components interact in complex ways. The identified serial mediation pathway is a novel contribution, emphasizing that a supportive environment acts as a critical bridge connecting knowledge and skills to behavioral intention and action (31). This chain of influence provides a more precise mechanism for understanding the persistent hand hygiene compliance challenges globally.

The present findings also carry cultural implications. Chinese healthcare settings are characterized by hierarchical relationships and strong peer influence (24), which may explain why opportunity and motivation exhibited stronger direct effects on behavior. This differs from individualistic Western contexts where personal capability may play a more direct role. Future cross-cultural comparisons could further elucidate these regional differences in hand hygiene behavior determinants. The implications of this study are both theoretical and practical. Theoretically, it provides robust empirical support for the COM-B model’s structure and its intricate mediating mechanisms within a Chinese healthcare context, thereby enriching the model’s application to specific health behaviors. Practically, the findings advocate for a paradigm shift away from singular, capability-focused campaigns toward integrated, system-oriented interventions. This entails a multi-faceted approach where hospital administrators must prioritize optimizing opportunity by ensuring the easy availability of alcohol-based hand rub and sinks, managing workloads to allow time for compliance, and fostering a unit culture with clear protocols and positive peer modeling (32). Concurrently, cultivating motivation requires sustained efforts through leadership endorsement, regular audit and feedback, and the establishment of peer reminder systems to strengthen social norms (33). Finally, capability building should be refined to evolve beyond basic knowledge, incorporating problem-solving skills that enable adherence to hand hygiene under real-world constraints, such as during high-workload periods (34).

However, translating these insights into sustained practice improvement faces well-documented challenges. The effectiveness of hand hygiene interventions often diminishes over time due to behavioral relapse, the waning of initial enthusiasm or audit effects (e.g., the Hawthorne effect), and competing clinical priorities (29, 33). Therefore, moving from short-term projects to enduring, system-integrated change is critical. This requires ongoing commitment from leadership, continuous resource allocation, and the embedding of hand hygiene support into the daily workflow, safety culture, and performance metrics of the healthcare organization. Sustainable strategies should plan for long-term maintenance, including regular refresher training, adaptive audit and feedback mechanisms, and leadership reinforcement of the desired norms.

## 5 Study limitations

This study has several limitations. First, the cross-sectional design precludes definitive causal inferences. Although structural equation modeling was employed to test hypothesized pathways, the temporal sequence implied by the mediation model cannot be conclusively established. Future longitudinal or experimental intervention studies are warranted to confirm the causal directions of the relationships proposed in the COM-B framework. Second, the reliance on self-reported data poses several measurement-related challenges. (a) Social desirability bias may have led to an overestimation of hand hygiene compliance and favorable perceptions of the COM-B constructs, as commonly observed in self-report studies (29, 30). The consistently high mean scores across all variables (ranging from 4.75 to 4.88) further suggest a potential ceiling effect and limited score variability, which may have constrained the detection of stronger relationships and limits the generalizability of the absolute score levels to settings with lower baseline compliance. (b) The very high correlations among the self-reported measures of capability, opportunity, and motivation, while theoretically plausible, raise concerns about common method variance and potential conceptual overlap in participants’ perceptions. Although confirmatory factor analysis supported the discriminant validity of the four-factor model, future research would benefit from complementing self-reports with more objective measures (e.g., direct observation of behavior, environmental audits for opportunity) to mitigate these biases. Third, while the revised structural equation model demonstrated acceptable fit on most indices, the elevated RMSEA (0.115) indicates a moderate overall fit. This suggests that other influential variables not specified in our model (e.g., organizational safety culture, specific workload metrics) may play a role (16). The RMSEA value may also be partly attributable to the large sample size (19). Future research could refine the model by integrating additional contextual or theoretical factors. Finally, the study was conducted in a single tertiary teaching hospital in China. The specific organizational culture, resources, and staff profile of this setting may limit the direct generalizability of the findings to other types of healthcare institutions (e.g., primary care clinics) or to other cultural contexts. Multi-center studies across diverse settings are needed to enhance the external validity of the results.

## 6 Conclusion

This SEM analysis confirms that the COM-B model provides a valid framework for understanding hand hygiene behavior among Chinese HCWs. Motivation and opportunity are the primary direct drivers of behavior. Capability, while essential, influences behavior predominantly through a positive indirect chain, by fostering a perception of opportunity which then boosts motivation. The significant negative direct effect of capability serves as a critical warning that investing solely in training without providing a supportive environment may be ineffective or even counterproductive. Therefore, sustainable improvement in hand hygiene compliance hinges on implementing multi-pronged strategies aimed at creating a synergistic system that simultaneously bolsters capability, optimizes opportunity, and cultivates motivation. This integrated approach is essential to bridge the “know-do” gap and ultimately achieve the fundamental goal of enhancing patient safety by reducing the burden of HAIs.

## Data Availability

The original contributions presented in the study are included in the article/Supplementary material, further inquiries can be directed to the corresponding author.
