# Supplementary material for: Capability, opportunity, and motivation: a structural equation model of hand hygiene behavior based on the COM-B framework in a Chinese hospital setting
Source: Front Public Health. 2026 May 8;14:1755052. doi: 10.3389/fpubh.2026.1755052 (PMC13194579; doi:10.3389/fpubh.2026.1755052)
Supplement: Supplementary file 1 [file Table_1.DOCX]

**Table S1** Complete modification indices (MI) for the four-factor CFA model

| **lhs** | **op** | **rhs** | **mi** | **epc** | **sepc.lv** | **sepc.all** | **sepc.nox** |
| --- | --- | --- | --- | --- | --- | --- | --- |
| Opp3 | ~~ | Opp4 | 688.711172064002 | 0.0657332872524492 | 0.0657332872524492 | 0.759231099183981 | 0.759231099183981 |
| Opp6 | ~~ | Opp7 | 684.999943762474 | 0.0393348695288538 | 0.0393348695288538 | 0.735190015303706 | 0.735190015303706 |
| Opp4 | ~~ | Opp6 | 439.749518718497 | -0.047684838 | -0.047684838 | -0.571435597 | -0.571435597 |
| Motivation | =~ | Opp6 | 362.019176000633 | 0.518606289796518 | 0.250307524097657 | 0.500047290452264 | 0.500047290452264 |
| Mot2 | ~~ | Mot3 | 355.931443958408 | 0.0119979770668417 | 0.0119979770668417 | 0.841544705804239 | 0.841544705804239 |
| Beh2 | ~~ | Beh3 | 353.784960206799 | 0.019808501810769 | 0.019808501810769 | 0.590223009132592 | 0.590223009132592 |
| Opp2 | ~~ | Opp5 | 327.650855613081 | -0.040111243 | -0.040111243 | -0.498597427 | -0.498597427 |
| Opp4 | ~~ | Opp5 | 319.828327942134 | 0.0559414161185423 | 0.0559414161185423 | 0.476175905687741 | 0.476175905687741 |
| Opp3 | ~~ | Opp6 | 317.910817180657 | -0.028672934 | -0.028672934 | -0.533016853 | -0.533016853 |
| Opportunity | =~ | Cap5 | 269.304876426222 | 0.597695841627275 | 0.350488130941942 | 0.5038115260125 | 0.5038115260125 |
| Opp4 | ~~ | Opp7 | 212.356993757427 | -0.034319746 | -0.034319746 | -0.398552722 | -0.398552722 |
| Motivation | =~ | Opp4 | 208.041847364086 | -0.617167927 | -0.297878716 | -0.412016928 | -0.412016928 |
| Beh1 | ~~ | Beh3 | 202.183932168658 | -0.021281525 | -0.021281525 | -0.416705198 | -0.416705198 |
| Mot4 | ~~ | Mot5 | 197.83797056032 | 0.0157293007810479 | 0.0157293007810479 | 0.382108251825497 | 0.382108251825497 |
| Opp6 | ~~ | Mot3 | 186.206661994049 | 0.0118011071131372 | 0.0118011071131372 | 0.390186090133816 | 0.390186090133816 |
| Opp4 | ~~ | Mot2 | 185.933742093831 | -0.016483285 | -0.016483285 | -0.419035574 | -0.419035574 |
| Cap5 | ~~ | Opp4 | 184.296767377253 | 0.0567908841598717 | 0.0567908841598717 | 0.343192890798217 | 0.343192890798217 |
| Opp5 | ~~ | Mot4 | 175.531060360575 | 0.0233175926668177 | 0.0233175926668177 | 0.346560071455637 | 0.346560071455637 |
| Cap3 | ~~ | Cap5 | 165.22739155472 | -0.025151197 | -0.025151197 | -0.459074098 | -0.459074098 |
| Opp3 | ~~ | Opp7 | 163.003035241031 | -0.021293848 | -0.021293848 | -0.383599193 | -0.383599193 |
| Motivation | =~ | Opp7 | 160.248662277523 | 0.357420832692806 | 0.172510679975289 | 0.329193805772142 | 0.329193805772142 |
| Cap5 | ~~ | Opp5 | 157.262332876459 | 0.0459080174140654 | 0.0459080174140654 | 0.317157755916018 | 0.317157755916018 |
| Beh1 | ~~ | Beh5 | 151.0524085 | 0.0246196980301654 | 0.0246196980301654 | 0.358541324188606 | 0.358541324188606 |
| Mot2 | ~~ | Mot4 | 149.414732876541 | -0.009561587 | -0.009561587 | -0.424422082 | -0.424422082 |
| Beh2 | ~~ | Beh5 | 144.675265999307 | -0.016932154 | -0.016932154 | -0.375238201 | -0.375238201 |
| Opp3 | ~~ | Mot3 | 136.842895788961 | -0.01093993 | -0.01093993 | -0.348630369 | -0.348630369 |
| Cap4 | ~~ | Cap5 | 134.257653658233 | 0.0275339745023482 | 0.0275339745023482 | 0.322203613286969 | 0.322203613286969 |
| Motivation | =~ | Opp3 | 133.726691151175 | -0.34818795 | -0.16805439 | -0.267945686 | -0.267945686 |
| Opp1 | ~~ | Opp6 | 131.867382785868 | -0.02424289 | -0.02424289 | -0.313726115 | -0.313726115 |
| Opp6 | ~~ | Mot2 | 129.287842862918 | 0.00868452693128455 | 0.00868452693128455 | 0.355331286572023 | 0.355331286572023 |
| Opp1 | ~~ | Opp4 | 121.638004486803 | 0.0365849903150499 | 0.0365849903150499 | 0.294163683017323 | 0.294163683017323 |
| Mot4 | ~~ | Beh5 | 121.173760128032 | 0.0144249579155867 | 0.0144249579155867 | 0.301370406668818 | 0.301370406668818 |
| Opp4 | ~~ | Mot4 | 113.777317812854 | 0.0214524820420358 | 0.0214524820420358 | 0.278897880863756 | 0.278897880863756 |
| Opp1 | ~~ | Opp7 | 109.069659250721 | -0.02283587 | -0.02283587 | -0.286377288 | -0.286377288 |
| Mot4 | ~~ | Beh3 | 108.92531369535 | -0.010198075 | -0.010198075 | -0.286466546 | -0.286466546 |
| Opp5 | ~~ | Beh3 | 105.46051607304 | -0.015280913 | -0.015280913 | -0.281042056 | -0.281042056 |
| Cap3 | ~~ | Mot2 | 101.875726894952 | 0.00529267820249489 | 0.00529267820249489 | 0.406393800656299 | 0.406393800656299 |
| Mot4 | ~~ | Beh1 | 101.106099352739 | 0.0166680305856652 | 0.0166680305856652 | 0.263744405115794 | 0.263744405115794 |
| Opp2 | ~~ | Mot4 | 96.4719067511822 | -0.013847208 | -0.013847208 | -0.262893495 | -0.262893495 |
| Cap3 | ~~ | Mot4 | 96.2825854530358 | -0.008551146 | -0.008551146 | -0.335781281 | -0.335781281 |
| Cap5 | ~~ | Mot2 | 96.0690623600746 | -0.014151607 | -0.014151607 | -0.291988477 | -0.291988477 |
| Mot2 | ~~ | Beh5 | 95.1654904754438 | -0.007680573 | -0.007680573 | -0.313775559 | -0.313775559 |
| Capability | =~ | Opp2 | 93.432798149481 | 0.229213803253901 | 0.123448369104309 | 0.215308117823717 | 0.215308117823717 |
| Cap3 | ~~ | Beh3 | 93.1431418885267 | 0.00711310577034149 | 0.00711310577034149 | 0.345635714957034 | 0.345635714957034 |
| Beh4 | ~~ | Beh5 | 91.7415983199046 | 0.012457374330196 | 0.012457374330196 | 0.391642331899721 | 0.391642331899721 |
| Opp1 | ~~ | Opp3 | 90.2118128498567 | 0.0221009019911648 | 0.0221009019911648 | 0.275662653092613 | 0.275662653092613 |
| Cap3 | ~~ | Beh1 | 89.8128183301167 | -0.011845409 | -0.011845409 | -0.324229575 | -0.324229575 |
| Mot2 | ~~ | Beh3 | 89.3563555965439 | 0.00554965823702017 | 0.00554965823702017 | 0.304832603795962 | 0.304832603795962 |
| Capability | =~ | Opp5 | 85.1405075767601 | -0.270507655 | -0.145688123 | -0.229645176 | -0.229645176 |
| Mot2 | ~~ | Beh1 | 84.0290533141314 | -0.009127598 | -0.009127598 | -0.282419665 | -0.282419665 |

Note. CFA = confirmatory factor analysis. The table lists all modification indices with MI > 10 from the initial four-factor CFA model. The columns represent the following: lhs = left-hand side variable; op = operator (e.g., '=~' for factor loading, '~~' for covariance); rhs = right-hand side variable; mi = modification index value; epc = expected parameter change; sepc.lv = standardized EPC on the latent variable level; sepc.all = completely standardized EPC. Based on theoretical plausibility, only covariances between error terms of items within the same construct (e.g., Opp3 ~~ Opp4) were freed in the revised model.
